# Do Traits Travel? Multiple-Herbicide-Resistant *A. tuberculatus*, an Alien Weed Species in Israel

**DOI:** 10.3390/plants12234002

**Published:** 2023-11-28

**Authors:** Idan S. Roth, Aviv Singer, Inon Yadid, Moshe Sibony, Zvi Peleg, Baruch Rubin

**Affiliations:** Robert H. Smith Institute of Plant Science and Genetics in Agriculture, Robert H. Smith Faculty of Agriculture, Food and Environment, The Hebrew University of Jerusalem, Rehovot 761000, Israel

**Keywords:** waterhemp, glyphosate, acetolactate synthase (ALS), protoporphyrinogen oxidase (PPO), gene transfer, seed-mediated gene flow

## Abstract

*Amaranthus tuberculatus* is the most common weed in soybean and corn in the USA and Canada. In Israel, it has been a minor riverbank weed. However, in recent years, growing densities of this plant have been observed in field crops, orchards, and roadsides. Between 2017 and 2022, we surveyed the distribution of *A. tuberculatus* and collected seeds for further study. We identified three main distribution zones in Israel: the Jezreel Valley, Hula Valley, and Coastal Plain. Most of the populations were found near water sources, fishponds, barns, dairies, or bird-feeding sites, suggesting the involvement of imported grain in introducing *A. tuberculatus* to Israel. Populations were screened for their responses to various post-emergence herbicides (i.e., ALS, EPSPS, PPO, HPPD, and PSII inhibitors). Several populations from the Jezreel Valley were found to be putatively resistant to ALS, EPSPS, and PPO inhibitors. The responses of those populations to trifloxysulfuron, glyphosate, and carfentrazone-ethyl were also studied. A single ALS-, EPSPS- and PPO-resistant plant was vegetatively propagated to create a clonal population, which was treated with foramsulfuron, glyphosate, and carfentrazone-ethyl. No resistance to PSII or HPPD inhibitors was observed, but resistance to herbicides that inhibit ALS, EPSPS, and PPO was observed. A clonal propagation assay revealed the existence of a population that was resistant to ALS, EPSPS, and PPO inhibitors. Since the local *A. tuberculatus* populations have not been exposed to herbicide selection pressure, these traits probably reached Israel through seed-mediated gene flow via imported grain.

## 1. Introduction

*Amaranthus tuberculatus* (Moq.) Sauer var. rudis (Sauer), also known as common waterhemp, is a dioecious, summer annual C_4_ weed with vigorous growth. The female plant can produce over 500,000 seeds [1], and dense populations of this weed can develop quickly [1,2]. The phenology of *A. tuberculatus* has been well-reviewed and reported [2,3,4]. Waterhemp has been declared the most common and the second most troublesome weed in soybean (*Glycine max* L. Merr.) fields in the USA [5], where this species has evolved resistance to various herbicidal modes of action (MOA), including multiple resistance [6].

The first herbicide-resistant (HR) *A. tuberculatus* was an ALS-resistant population reported in Illinois, USA, in 1993 [6]. Later, populations with resistance to different MOAs were detected. However, the distribution of these HR populations was limited to the US (Iowa, Minnesota, Missouri, Kansas, Nebraska, etc.) [6]. In 2002, a population from Ontario, Canada, was also found to be ALS- and PSII-resistant [6]. That was the first report of HR waterhemp outside the USA. Yadid et al. reported in 2017 an ALS-resistant population of *A. tuberculatus* in row crops in Israel, such as corn (*Zea mays* L.), cotton (*Gossypium hirsutum* L.), and sunflower (*Helianthus annuus* L.) [7]. Later, another ALS-resistant population was reported in Italy [8,9]. The authors of those studies proposed that the populations they had encountered could have originated from North America. These first reports of HR *A. tuberculatus* outside of North America stand as milestones in disseminating this species as a troublesome herbicide-resistant weed.

In Israel, *A. tuberculatus* is an alien weed species. It was first found in 1970 on the banks of the Jordan River and, in 1982, was identified as *A. rudis* Sauer [10]. However, in recent years, we have detected the weed in increasing densities in different regions of the country near animal-feed compounds. Simultaneously, waterhemp had become more noticeable in row crops, orchards, and roadsides. Failure to control this weed has been observed following the application of herbicides, such as ALS inhibitors, PPO inhibitors, and glyphosate, indicating the possible existence of putative HR populations. Furthermore, it is very easy for the existing population to spread from one field to another via agricultural machinery. Shattered seeds are probably dispersed via soil and water erosion and/or rivers and streams, introducing them to new sites [11]. This worrying increased infestation calls for a study of this weed’s biology and its responses to different herbicides to ensure proper management. This study aimed to (i) conduct a survey detailing the distribution of *A. tuberculatus* in Israel and (ii) examine the responses of those different populations to ALS, EPSPS, PSII, HPPD, and PPO inhibitors.

## 2. Results

### 2.1. Distribution of A. tuberculatus: Survey Results

**Figure 1:**
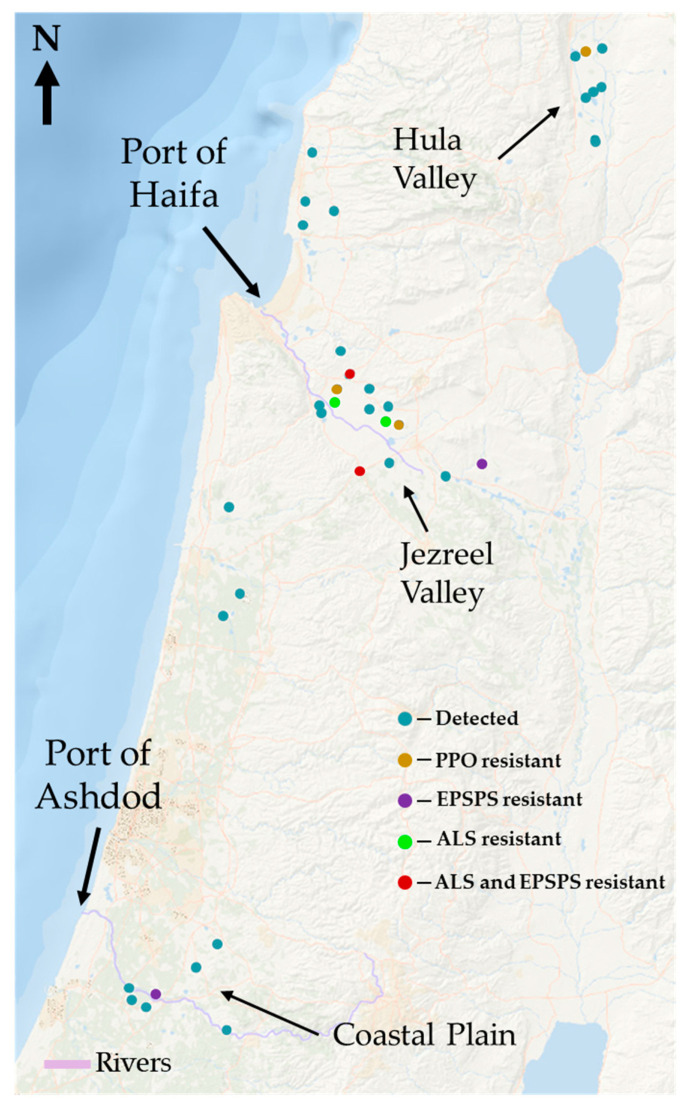
Map of the distribution of *A. tuberculatus* in Israel. This species has been found in three main areas: the Jezreel Valley, the Hula Valley, and the Coastal Plain. Detected = populations from which seeds were not collected or populations identified as susceptible.

We randomly detected and sampled 37 *A. tuberculatus* populations in three main regions: the Jezreel Valley, the Hula Valley, and the Coastal Plain, as well as two minor areas, the Western Galilee and the Sharon Plain (Figure 1). Initially, most populations were found at non-cultivated sites, such as roadsides and near cowsheds, the banks of fishponds, regional animal-feed production sites, and along riverbanks. However, during the last few years, perhaps due to anthropogenic activities, more populations have been detected and collected from infested irrigated summer crops, such as cotton, corn, sunflower, and olive groves, particularly in the Jezreel Valley.

### 2.2. Herbicide Screening

**Figure 2:**
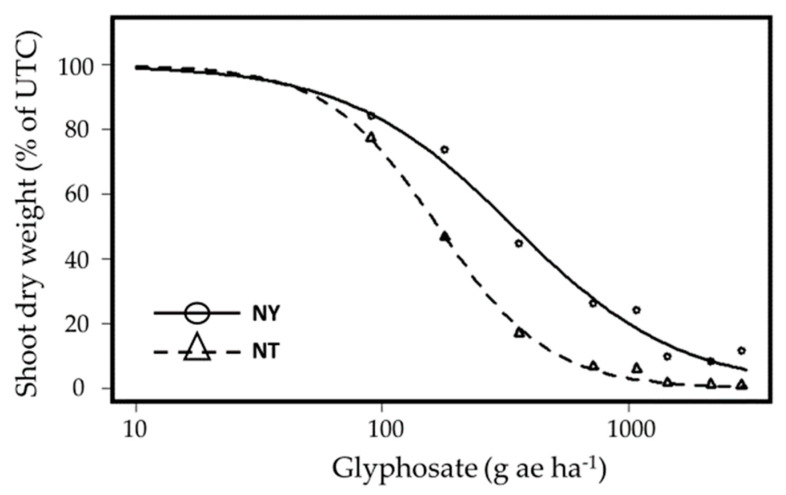
Dose-response curves of two *A. tuberculatus* populations, Newe Ya’ar (NY, R) and Nahal Timnah (NT, S), to glyphosate (0, 90, 180, 360, 720, 1080, 1440, 2160, 2880 g acid equivalent (ae) ha^−1^) applied post-emergence at the four-to-six-leaf stage. The data represent the mean shoot dry weights (% of untreated control) of three different experiments. Plants were harvested 21 days after application (DAA). UTC-untreated control.

**Figure 3:**
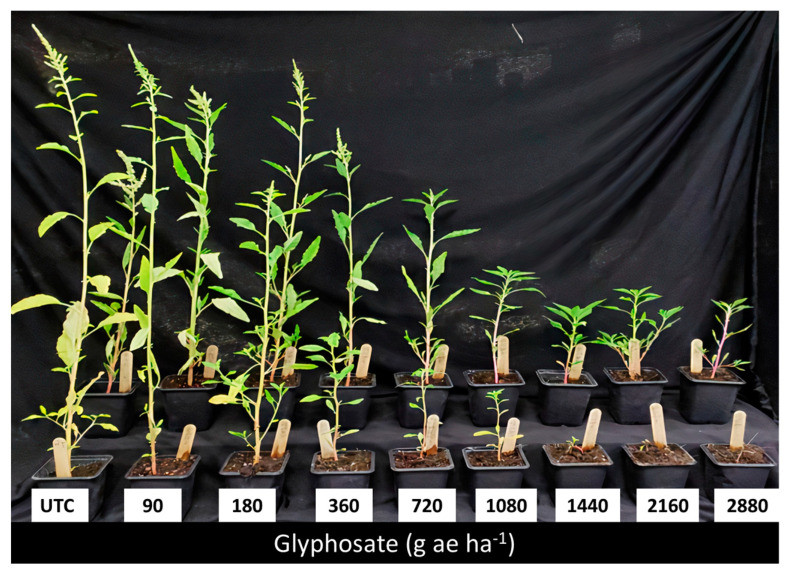
Glyphosate dose responses (21 DAA) of two populations of *A. tuberculatus* at 21 DAA, Newe Ya’ar (back row, resistant) and Nahal Timnah (front row, susceptible). The numbers on the pots indicate glyphosate doses of 0, 90, 180, 360, 720, 1080, 1440, 2160, and 2880 g ae ha^−1^. The recommended dose is 720 g ae ha^−1^.

**Figure 4:**
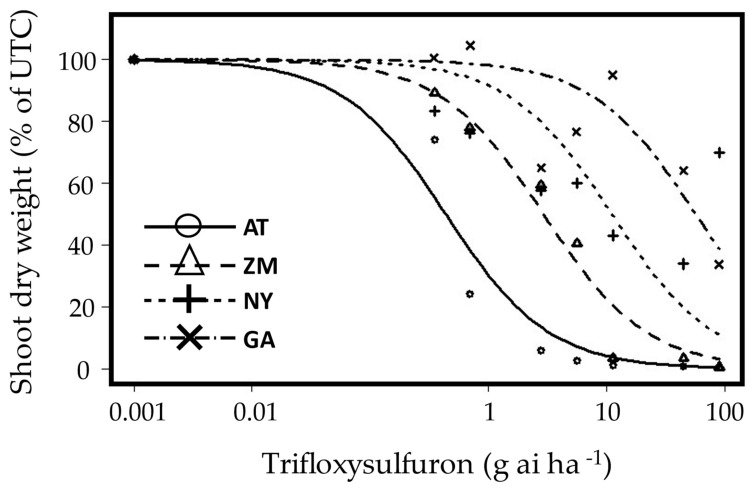
Dose–response curves of four *A. tuberculatus* populations: (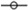) AT-Tel Nof, (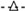) ZM-Tzora, (ALS-intermediate), (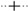) NY-Newe Ya’ar and (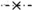) GA-Ginegar (ALS-resistant). Trifloxysulfuron (0, 0.35, 0.7, 2.8, 5.6, 11.3, 45 and 90 g ai ha^−1^) was applied at the four-to-six-leaf stage. The data represent the mean shoot dry weights (% of control) of plants harvested at 21 DAA. Plants were grown in a net-house under ambient summer conditions. UTC-untreated control.

**Figure 5:**
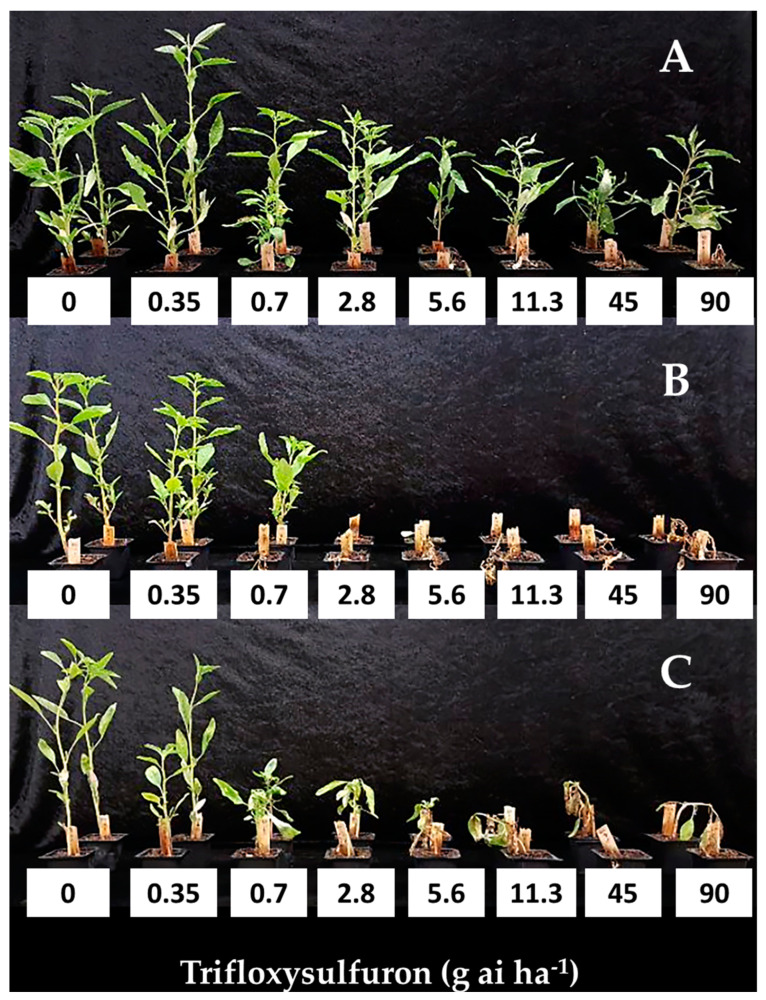
Trifloxysulfuron dose responses (21 DAA) of three *A. tuberculatus* populations: (**A**)-Newe Ya’ar (resistant), (**B**)-Ginegar (resistant) and, (**C**)-Tel Nof (susceptible). The pots are labeled with the applied trifloxysulfuron doses: 0, 0.35, 0.7, 2.8, 5.6, 11.3, 45 or 90 (g ai ha^−1^). 11.3 g ai ha^−1^ is the recommended dose.

Among the 37 collected populations, four of the examined populations were confirmed as ALS-resistant, three as PPO-resistant, and four as EPSPS-resistant (Table 1). All examined populations were susceptible to aclonifen, fomesafen, and flumioxazin (PPO-inhibiting herbicides), and tembotrione (HPPD-inhibiting herbicides). Some of the populations were also resistant to more than one MOA (multiple resistance), specifically the population from Newe Ya’ar, which was resistant to both trifloxysulfuron and glyphosate (Figure 2, Figure 3, Figure 4 and Figure 5) and the population from Megiddo that was resistant to PPO, EPSPS, and ALS inhibitors. Some populations were categorized as resistant due to their ability to survive a double dose of herbicide but had no dose-response curves constructed for them; those populations are marked accordingly in Figure 1. Our screening did not reveal populations with resistance to MOAs other than inhibitors of EPSPS, ALS, and PPO, although some survival was observed following the application of atrazine (Table 1).

### 2.3. Glyphosate Resistance

The response of the putative glyphosate-resistant (GR) populations to the herbicide was examined three times during the Israeli summer using two populations: Newe Ya’ar (GR) and Nahal Timnah-glyphosate susceptible (GS). The response of each experiment to glyphosate was quite similar, enabling a combined analysis, and is presented in Figure 2. The ED_50_ values were 341.5 ± 28.6 g ae ha^−1^ and 168.2 ± 11.8 g ae ha^−1^ for the GR and GS populations, respectively. The resistance index (RI) (i.e., the ratio between the ED_50_ value of the resistant (R) compared to that of the susceptible (S) population (R/S)), in this case was relatively low for *A. tuberculatus* (Table 2) compared to other reported cases. In the literature, we mainly find reports of ~ 5–10-fold resistance indices [12,13], whereas we report an RI of ~2. RI is indeed an important tool for quantifying the level of resistance. However, this value is just as dependent on the response of the susceptible population as it is on the resilience of the resistant population. In addition, these populations are in transition and, being "newcomers", may contain a mixture of GR and GS individual plants, a fact that increases the variability within each population.

### 2.4. ALS Resistance

Following the screening (Table 2), four populations were chosen for further inspection. Dose-response curves (Figure 4 and Figure 5) were constructed for Newe Ya’ar and Ginegar populations from the Jezreel Valley (putative ALS-resistant) and Tzora and Tel Nof (putative ALS-susceptible), from the Coastal Plain. ED_50_ values for the resistant populations were 11.11 and 26.85 g ai ha^−1^ trifloxysulfuron, for Newe Ya’ar and Ginegar, respectively. ED_50_ value for the susceptible population from Tel-Nof was 0.43 g ai ha^−1^_._ The population from Tzora varied in its response to trifloxysulfuron, where some plants were killed. In contrast, others were injured, resulting in an ED_50_ value of 2.92 g ai ha^−1^ which is significantly higher than the ED_50_ of the apparent susceptible Tel-Nof population, but significantly lower than that of the resistant populations from Newe-Ya’ar and Ginegar, hence it was designated in Figure 4 as intermediate (I). RI values were 6.8, 25.8-fold, and 132.3 folds for Tzora, New-Ya’ar, and Ginegar, respectively, compared to the Tel-Nof sensitive population (Table 2).

### 2.5. PPO Resistance

**Figure 6:**
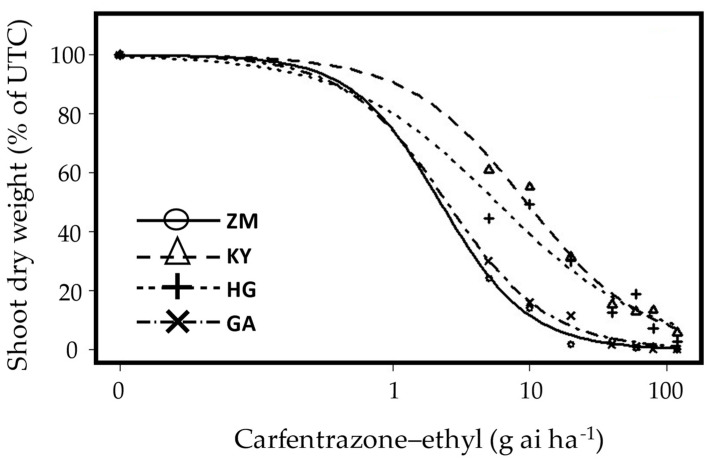
Dose–response curves of four *A. tuberculatus* populations from the Jezreel Valley: carfentrazone-ethyl-susceptible-(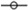) ZM-Tzora; (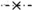) GA-Ginegar; carfentrazone-ethyl-resistant (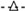) KY-Kfar Yehoshua and (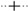) HG-Havat Gadash. Carfentrazone-ethyl (0, 5, 10, 20, 40, 60, 80, and 120 g ai ha^−1^) was applied post-emergence to seedlings at the four-to-six-leaf stage, which were grown in a net-house under ambient summer conditions. The experiment was repeated twice, and the data represent the mean shoot dry weights (% of control) of plants harvested at 21 DAA. UTC-untreated control.

**Figure 7:**
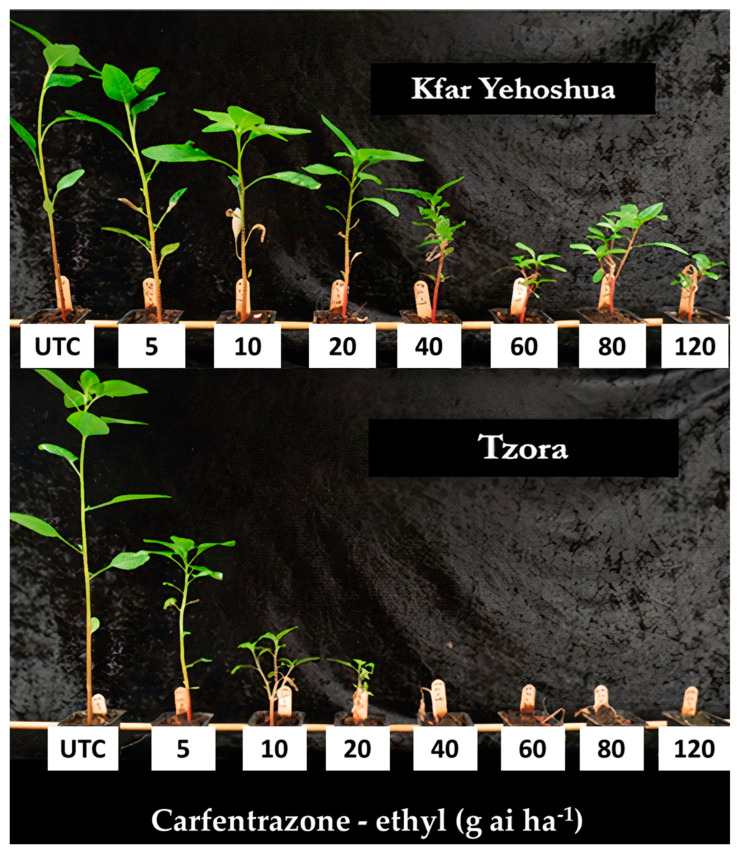
Carfentrazone-ethyl dose responses (21 DAA) of two populations of *A. tuberculatus*: Tzora (susceptible) and Kfar Yehoshua (resistant). Numbers indicate doses of 0, 5, 10, 20, 40, 60, 80, and 120 g ai ha^−1^ applied post-emergence to seedlings at the four-to-six-leaf stage, which were grown in a net-house under ambient summer conditions. The recommended dose is 20 g ai ha^−1^.

Two populations were confirmed as carfentrazone-ethyl-resistant using dose-response curves (Figure 6 and Figure 7). A few more populations survived the recommended rates of the herbicides (Table 1) and the population from Megiddo (Jezreel Valley) even survived two more PPO inhibitors (oxyfluorfen and oxadiazon; Table 1). In this case, rapid injury was evident following exposure to carfentrazone-ethyl, which is apparently similar to the phoenix phenomenon described for EPSPS resistance [14], as the resistant population was injured but then recovered.

### 2.6. Multiple Herbicide Resistance

**Figure 8:**
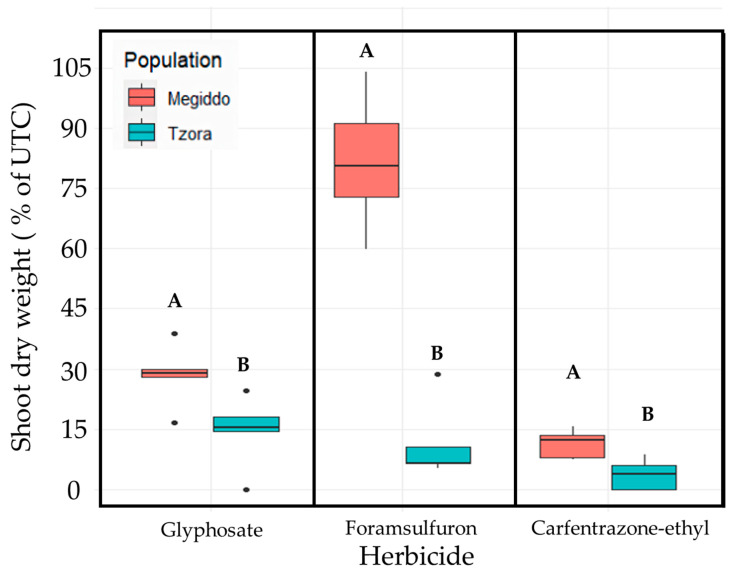
Results of a multiple-resistance assay using clonal propagated plants. Plants were propagated from a single mother plant from each population (Megiddo-resistant and Tzora-susceptible). The plants were treated post-emergence with carfentrazone-ethyl (20 g ai ha^−1^), foramsulfuron (45 g ai ha^−1^), and glyphosate (360 g ae ha^−1^). The data represent the mean shoot dry weights (% of control) of plants harvested at 21 DAA. The responses of the different populations were tested twice, and the means were compared using a student’s *t*-test for each herbicide treatment. Means followed by the same letter are not significantly different (*p* ≤ 0.05). UTC-untreated control.

The multiple-resistance assay (Figure 8) showed that the clonal propagated cuttings from the Megiddo population (R) withstood the application of all three herbicides (glyphosate (EPSPS), foramsulfuron (ALS) and carfentrazone-ethyl (PPO)), while the susceptible clonal cuttings from the Tzora population (S) were significantly injured following the treatments.

## 3. Discussion

### 3.1. Distribution of A. tuberculatus

Although HR *A. tuberculatus* populations were reported in Israel (Asia), Italy (Europe), and recently in Uruguay (South America), the vast majority of HR *A. tuberculatus* cases were recorded in North America where, in a short period, it became one of the most troublesome HR weeds in soybean and corn [5,6]. In addition to harboring a genetic pool of HR *A. tuberculatus,* the USA is also a major exporter of grains (second and fifth biggest exporter of corn and wheat, respectively [15]). Thus, the outflow of *A. tuberculatus* seeds with or without HR traits via contaminated shipments of goods is plausible, as documented in Europe and the Mediterranean countries [16].

The unique distribution of *A. tuberculatus* in Israel indicates at least three different invasion events, presumably via imported grain shipments, with further dispersal powered by anthropogenic activities, as recently described for other invasive species in China [11,17]. The first invasion event was recorded in the Hula Nature Reserve, a habitat for water birds in the Hula Valley that currently serves as a crane (*Grus grus*) feeding site and is heavily infested with *A. tuberculatus*. Cranes migrate from cold northern countries to spend the winter in the warm Hula Valley, where they feed on fish and crops, particularly peanuts (*Arachis hypogaea* L.), grown in and around the reclaimed muck soils of the Hula Lake. Starting in the early 2000s, farmers have been importing corn grains to feed the wintering cranes in order to draw them away from the cultivated crops, hence minimizing this heavy damage to crops [18]. The first detection of *A. tuberculatus* was monitored in 2009 at the feeding site and at 2018 it was a major invasive weed in this Natural Reserve [19]. The Hula Nature Reserve is probably the primary source of other *A. tuberculatus* populations detected in 2018 and 2019 in the Hula Valley (Figure 1).

**Figure 9:**
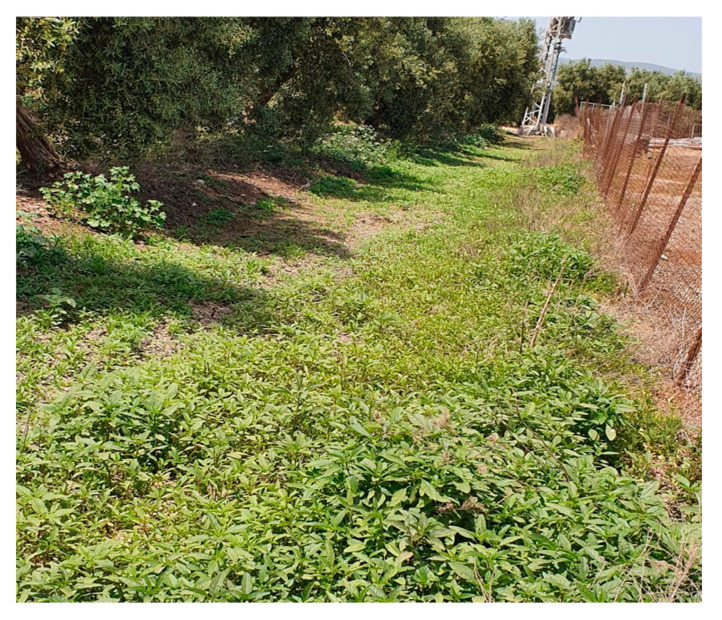
*Amaranthus tuberculatus* infestation following local dairy cow manure application in olive grove-Jezreel Valley (2018).

The second possible introduction region is the Jezreel Valley along the Kishon River, where most of the detected populations were identified as HR within crops and around dairy sheds. This region is characterized by fertile, heavy soils (50–60% clay) and various irrigated, well-fertilized summer row crops-an appropriate habitat for *A. tuberculatus* proliferation [3]. Moreover, heavy *A. tuberculatus* infestation was observed in olive groves following the application of fresh local dairy manure (Figure 9). Another possible introduction site, where most populations detected are herbicide-sensitive, is the area surrounding the Sorek River, which runs along the Coastal Plain. Despite the similarities to the Jezreel Valley in terms of soil types and irrigated crop selection, much lower infestation was observed, possibly due to the fact that being herbicide-sensitive limited their distribution to riverbanks, field margins, and roadsides.

### 3.2. Glyphosate Resistance

It should be mentioned that the current study is the first report of GR *A. tuberculatus* outside North America. Despite the low RI values observed, which can be attributed to the high ED_50_ value of the susceptible population, the GR plants survived and set seeds following four folds of glyphosate recommended rates (Figure 3), thus potentially increasing the GR distribution. In some plants, the phoenix phenomenon (in which a plant is severely injured by an herbicide but regrows and sets seed) was visible, indicating that the resistance mechanism might, at least in part, be related to a non-target-site mechanism, where insufficient amount of herbicide reaches the target site [20]. It should be noted that genetically engineered crops (e.g., Roundup Ready^®^ crops) are not registered or used in Israel. Despite this fact, glyphosate resistance has previously been reported in Israel in *Lolium rigidum* [21] and *Conyza bonariensis* [22,23], but not in any of the other *Amaranthus* species growing in the country (i.e., *A. palmeri*, *A. retroflexus*, *A. spinosus*, *A. albus*, *A. blitoides*, *A. graecizans*, *A. viridis*, *A. cruentus*, and *A. blitum*). This increases the risk of gene flow via inter-species hybridization [24].

In addition, as a recent invader, *A. tuberculatus* still has a limited distribution in the country and has hardly been exposed to herbicide treatments that could impose a shift toward individual herbicide-resistant plants. Hence, we assume that the detected herbicide-resistant populations were probably imported through contaminated animal-feed grain shipments, most probably from a country where GR *A. tuberculatus* was previously reported.

### 3.3. ALS Resistance

High RI values (>25) detected in the populations from the Jezreel Valley are usually attributed to an altered ALS target-site mechanism, whereas lower RI values, as detected in the population from the Coastal Plain (5–10), are usually related to a non-target site resistance mechanism [1,25]. Studies are in progress to elucidate the mechanism rendering the observed resistance. Since ALS-inhibiting herbicides are widely used, along with the exogamous nature of *A. tuberculatus*, one can expect a rapid dissemination of this trait in the region.

Despite comparable cultivation of winter and summer crops in the Jezreel Valley and the Coastal Plain, all the ALS-resistant populations were collected exclusively from the Jezreel Valley (Figure 1). In contrast, the populations from the Coastal Plain were either ALS-susceptible or ALS–intermediate, indicating that the latter population might be in transition. Furthermore, the Haifa Port (Figure 1), which is close to the Jezreel Valley, and the Ashdod Port, which is close to the Coastal Plain region, both serve for unloading and delivery of imported grains to adjacent feed-production centers, dairy farms, and fishponds, possibly linking the detected *A. tuberculatus* populations with entry ports, as was reported for common ragweed in China [17]. These findings support our hypothesis that there were several independent introductions of this *A. tuberculatus*.

That said, ALS resistance is known to evolve rapidly [1,6,25]. Therefore, local evolution of this trait is conceivable, while other resistance traits are less likely to evolve quickly post-invasion. Interestingly, the anticipated even distribution of ALS–resistant *A. tuberculatus* across regions was not observed.

### 3.4. PPO Resistance

The MOA and resistance mechanism of PPO-inhibiting herbicides were recently reviewed [26]. In the study, the authors indicate that most PPO-resistant cases were found mainly in the US, showing *A. tuberculatus* as the most prevalent resistant weed species. To the best of our knowledge, this is the first report of PPO-resistant *A. tuberculatus* outside North America [6]. The resistant populations were detected mainly in the Jezreel Valley, where carfentrazone-ethyl is not commonly used in irrigated row crops.

PPO-resistance takes time to evolve, since the first case of PPO-resistant was *A. tuberculatus* in Kansas in 2001 [27], well after this MOA was commercialized [26], whereas, in our case, the carfentrazone-ethyl resistant populations were detected with no apparent prior exposure to herbicide. This further supports our hypothesis that *A. tuberculatus* seeds invaded Israel, already carrying herbicide-resistance traits. Interestingly, despite the considerable resistance level observed (RI of 9.4 and 5.7), most of the populations exhibited resistance to carfentrazone-ethyl only, as reported by Obenland et al. from Illinois [28]. These results indicate the involvement of a non-target site resistance mechanism. Known mutations, such as the deletion of glycine at position 210 (ΔG210) or the substitution of arginine at position 128, endow resistance to most known PPO-inhibiting herbicides yielding significantly high resistance indices (<25 fold) [1,26,28,29,30].

Fortunately, the resistant populations were only resistant to carfentrazone-ethyl (triazolinones), allowing the future use of PPO inhibiting herbicides, an important weed management tool of *A. tuberculatus* in infested fields.

### 3.5. Multiple Herbicide Resistance

Multiple-herbicide-resistant *A. tuberculatus* is not an uncommon scenario. To date, there are 23 unique cases of multiple-herbicide-resistant (MHR) *A. tuberculatus,* all of which are reported in North America [6]. The discovery of the MHR populations to ALS-, PPO- and EPSPS-inhibiting herbicides in the Jezreel Valley, a region where most of the HR populations were found, is consistent with the unique distribution of the species in Israel. The presence of MHR populations may indicate that the introduction of *A*. *tuberculatus* is not a singular event but an ongoing phenomenon. This weed was first detected in Israel around riverbanks in 1970 [10], long before the first reported MHR *A. tuberculatus* in 2006 in the US [6]. The expansion of these traits outside North America to new regions further prompts the consideration of ongoing seed-mediated gene flow via grain imports to the region.

*A. tuberculatus* can now be added to the list of MHR weeds reported locally: *A. blitoides*, *A. palmeri*, *L. rigidum*, *C. canadensis*, and *Senecio vernalis* [6,21,22,23]. This disturbing distribution of MHR species threatens the sustainability of the agro-system, as most of these herbicides are widely used on local farms. The ongoing effort of regulators to ban the use of different herbicides and the lack of new MOAs on the horizon leaves farmers with minimal options for chemical weed management.

## 4. Materials and Methods

### 4.1. Survey and Plant Material

Seeds of *A. tuberculatus* were collected from 2017 to 2021 from plants detected in ruderal sites or cultivated fields. Seeds collected from several plants in one site are defined as a population. The location and collection date of each population were recorded and mapped using Google Maps. The seeds were dried at 35 °C for one week, threshed (Test Sieve Ari Levy, LTD), and stored at 4 °C until used. For each experiment, seeds were sown in pots (9 × 9 × 9.5 cm or 6 × 6 × 7 cm) containing a commercial potting mix (Tuff Marom Golan, Israel). Seedlings were grown in a net-house under ambient summer conditions or controlled conditions in a phytotron under natural daylight at temperatures of 28/22 °C (16 h day/ 8 h night) and irrigated as needed.

### 4.2. Herbicide-Response Screening

Application of herbicides was conducted using a 4th Generation Research Chamber Sprayer (DeVries Manufacturing Inc., Hollandale, MN, USA) equipped with an 80015E nozzle delivering a 200 L ha^−1^ spray volume.

A preliminary examination of the collected *A. tuberculatus* populations (screening assay) was performed on plants grown as described in Section 4.1. Herbicides with different MOAs were applied post-emergence at five replicates, as described earlier, on seedlings at the 4–6 leaves stage. Herbicides and doses used are given in Table 3. *A. tuberculatus* populations exhibiting both a survival rate of >40% and a shoot dry biomass of >25% relative to the control were identified as putative-resistant populations. Plant shoots were harvested 21 days after application (DAA), oven-dried at 70 °C for 72 h, and shoot dry weight was recorded. All experiments were repeated at least twice. Glyphosate, trifloxysulfuron and carfentrazone-ethyl were selected for further studies in recognition of their frequent use locally and the results of the screening assay.

### 4.3. Dose–Response Studies

*A. tuberculatus* plants from Newe Ya’ar (putative GR) and Nahal Timnah (GS) populations were used for the dose–response studies. Plants were grown and sprayed as described in Section 4.1 and 4.2 at 4 to 6 leaves stage with glyphosate (Roundup^®^, 360 g ae glyphosate L^−1^ as isopropylamine salt (Bayer Agriculture BVBA, Belgium) at rates of 0, 90, 180, 360, 720, 1080, 1440, 2160 and 2880 g ae ha^−1^. Plants were then grown in a net-house under ambient summer conditions. The experiment was repeated three times with 6 to 10 replicates per each treatment. Data represent the mean shoot dry weights of the three experiments as % of untreated control.

*A. tuberculatus* plants from Newe Ya’ar and Ginegar (putative ALS resistance) and Tzora and Tel Nof (putative ALS-susceptible) populations were grown and sprayed (as described in Section 4.1 and Section 4.2) at 4–6 leaves stage, with five replicates per treatment with trifloxysulfuron (Envoke^®^ WG 75% trifloxysulfuron, Syngenta, Basel, Switzerland) at 0, 0.35, 0.7, 2.8, 5.6, 11.3, 45 and 90 g ai ha^−1^. The experiment was conducted twice in a similar manner but the second run of the experiment included additional application rates (i.e., 1.4 and 22.5 g ai ha^−1^).

*A. tuberculatus* plants from Tzora and Ginegar (PPO susceptible) and from Havat Gadash and Kfar Yehoshua (putative PPO resistance) populations were grown and sprayed (as described in Section 4.1 and Section 4.2) at 4–6 leaves stage with carfentrazone–ethyl [Spotlight^®^ OD, 60 g carfentrazone-ethyl L^−1^, FMC, USA] at rates of 0, 5, 10, 20, 40, 60, 80, and 120 g ai ha^−1^. Experiments were run twice with five replicates, and shoot dry weight was recorded as described in Section 4.3.

### 4.4. Clonal Cutting: Multiple Herbicide Resistance Assay

**Figure 10:**
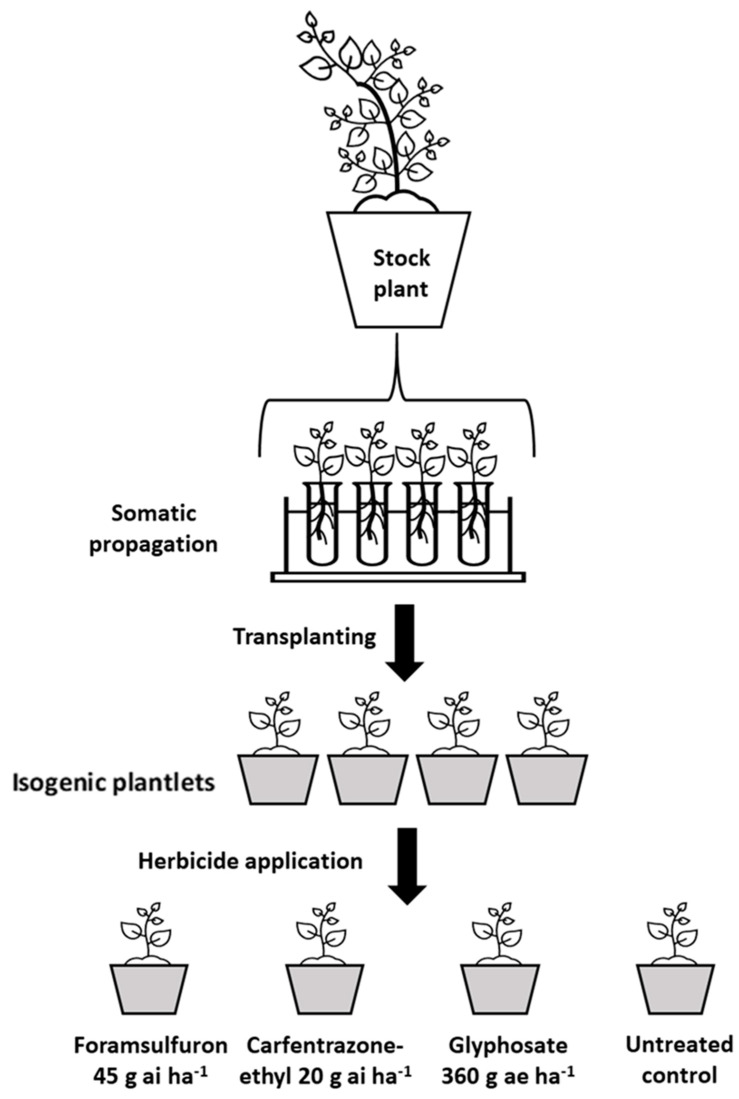
Schematic description of the clonal cutting experiment. Five stock plants from two *A. tuberculatus* were examined. Four cuttings were rooted in water from each stock plant, transferred to a growth mix in pots, and treated with the different herbicides in five biological replicates.

In order to demonstrate the presence of multiple herbicide resistance at the single plant level, stock plants from two populations, Megiddo (R) and Tzora (S), served as parent plants in five replicates. The plants were grown in 3L pots until branching. Small, 7-10-cm-long twigs (cuttings) with two to four leaves each, were excised from each single parent plant, inserted into 25-mL glass flasks containing water, and allowed to develop roots. Rooted plantlets were transplanted into pots (9 × 9 × 9.5 cm) containing potting mix and grown for one week in a controlled environment, as described above (Section 4.1). One plantlet from every single parent was treated post-emergence either with foramsulfuron, carfentrazone-ethyl or glyphosate applied at 45, 20, and 360 g ai ha^−1^, respectively, as described above (Section 4.2). In this way, isogenic plantlets originating from the same plant were treated with multiple herbicides (Figure 10). Plant shoot dry weight was recorded at 21 DAA and compared to untreated control shoot dry weight.

### 4.5. Data Analysis

Data analyses were performed using R programming software (version 4.2.3) [31]. Dose-response analysis was conducted by fitting a two-parameter log-logistic function with the upper, using the ‘DRC’ package [32]. The model estimates the log-logistic equation:

$$f\left(x\right)=\frac{1}{1+e^{\left(b\left(\log\left(x\right)-\log\left(e\right)\right)\right)}}$$

where *f(x)* is shoot dry biomass % of untreated control, *x* is the herbicide rate (g ai/ae ha^−1^), *b* is the slope at the infliction point, and *e* is ED50; g ai/ae ha^−^^1^ (i.e., The herbicide dose that caused a 50% reduction in biomass). Box-plots were constructed using the GGPLOT 2 package [33] and means were compared using a student’s *t*-test (*p* ≤ 0.05) between the two population within each herbicide.

## 5. Conclusions

The eco-geographic distribution of this species in three regions indicates that *A. tuberculatus* has invaded Israel probably via multiple entry points: along the Kishon River in the Jezreel Valley, along the Sorek River in the Coastal Plain, and at the crane-feeding site in the Hula Valley (Figure 1). This hypothesis is supported by observations of the specific sites at which the populations were first detected—in regional feed-production centers, dairy houses, and a crane-feeding site—as well as their proximity to rivers and ports where imported grain shipments are unloaded. It should be noted that some populations collected in the Jezreel and Hula Valleys were identified as highly resistant to ALS inhibitors, other populations were identified as GR and others exhibited resistance to carfentrazone. In addition, MHR was also confirmed for three different MOAs. In contrast, most populations collected in the other regions were diagnosed as herbicide-susceptible. One can assume that, due to the lack of regulation, inadvertent importation of weed seeds is ongoing, a situation that, together with anthropogenic activities, facilitates multiple introductions and wider dispersal of the weed carrying alien traits.

The above supports the hypothesis that *A. tuberculatus* seed reached Israel with herbicide-resistant traits via seed-mediated gene flow. Further research is underway to uncover the mechanisms rendering the observed herbicide resistance.

## Figures and Tables

**Table 1:** Responses of *A. tuberculatus* populations to herbicides with different modes of actions applied at a rate given in the Materials and Methods. Shoot dry weight (DW) and plant survival are expressed as % of untreated control. Means marked with an asterisk (*) are significantly different from the respective susceptible population analyzed using Student’s *t*-test (*p* ≤ 0.05). Populations exhibiting a survival rate of >40% and/or shoot dry biomass of >25% relative to the control identified as putative-resistant populations. Population exhibiting survival below 40% and <25% shoot DW were considered susceptible. NA = not measured.

Population	Protoporphyrinogen oxidase (PPO) inhibitors									
	Carfentrazone-ethyl		Oxyfluorfen		Oxadiazon		Sulfentrazone		Pyraflufen-ethyl	
	Shoot DW	Survival	Shoot DW	Survival	Shoot DW	Survival	Shoot DW	Survival	Shoot DW	Survival
	% of untreated control									
Tzora (S)	0.0	0.0	0.0	0.0	9.3	50.0	0.0	0.0	0.0	0.0
Havat Gadash	22.4 *	66.6	14.1	100.0	25.1	100.0	2.1	16.6	9.9 *	50.0
Newe Ya’ar	2.3	16.6	6.4	33.3	6.2	50.0	0.0	0.0	0.0	0.0
Megiddo	59.7 *	100.0	28.4 *	66.6	42.3 *	83.3	0.4	16.6	3.1	16.6
Kfar Yehoshua	19.4 *	66.6	0.8	16.6	8.0	50.0	0.0	0.0	0.0	0.0
Ginegar	3.2	33.3	12.9	83.3	18.8	100.0	0.0	0.0	2.5	16.6
Nahal Timnah	0.0	0.0	0.0	0.0	8.2	66.6	0.0	0.0	0.0	0.0
Population	PSII inhibitor		Acetolactate synthase (ALS) inhibitors				EPSPS inhibitor			
	Atrazine		Trifloxysulfuron		Pyrithiobac-sodium		Glyphosate			
	Shoot DW	Survival	Shoot DW	Survival	Shoot DW	Survival	Shoot DW	Survival		
	% of untreated control									
Hulata	0.2	0.0	NA	NA	NA	NA	NA	NA		
Havat Gadash (S)	6.0	40.0	0.1	0.0	0.1	0.0	0.0	0.0		
Tzora	0.2	80.0 *	5.8	20.0	0.2	0.0	2.7	20.0		
Newe Ya’ar	19.1	80.0 *	48.1 *	80.0 *	72.4 *	80.0 *	27.3 *	100.0 *		
Megiddo	26.3	100.0	108.5 *	100.0 *	64.6 *	100.0 *	29.3 *	80.0 *		
Kfar Yehoshua	36.1	80.0 *	65.2 *	80.0 *	60.7 *	60.0 *	46.7 *	100.0 *		
Ginegar	6.0	40.0	110.7 *	100.0 *	88.0 *	100.0 *	9.4	60.0		

**Table 2:** Responses of the different populations to the PPO, ALS, and EPSPS herbicides, along with their ED_50_ values and resistance-index (RI) values. R = resistant; S = susceptible, I = intermediate. Values were extracted as described in Materils and Methods.

Herbicide (MoA)	Population	Response	ED_50_ (g ai ha^−1^)	RI
Glyphosate (EPSPS)	Nahal Timnah	S	168.2 ± 11.8	--
	Newe Ya’ar	R	341.5 ± 28.6	2.0
Trifloxysulfuron (ALS)	Tel-Nof	S	0.4 ± 0.17	--
	Tzora	I	2.9 ± 1.0	6.8
	Newe-Ya’ar	R	11.1 ± 6.3	25.8
	Ginegar	R	56.8 ± 24.4	132.3
Carfentrazone-ethyl (PPO)	Tzora	S	2.2 ± 1.4	--
		R	9.5 ± 1.8	4.3
	Havat Gadash	R	5.8 ± 1.7	2.6

**Table 3:** Herbicide active ingredients (ai) or acid equivalent (ae) for different modes of action, were applied at the recommended dose to *A. tuberculatus* populations post-emergence at the 4–6-leaf stage.

Mode of Action *	Active Ingredient	Dose (g ai/ae ha^−1^)
ALS	Trifloxysulfuron	11.3
	Pyrithiobac–sodium	51.7
PPO	Oxyfluorfen	480
	Carfentrazone–ethyl	20
	Sulfentrazone	336
	Pyraflufen	7.2
	Oxadiazon	500
	Aclonifen	600
	Flumioxazin	40
	Fomesafen	200
EPSPS	Glyphosate	1080
HPPD	Tembotrione	99
PSII	Atrazine	500

* Acetolactate synthase (ALS), protoporphyrinogen oxidase (PPO), 5-enolpyruvylshikimate-3-phosphate synthase (EPSPS), 4-hydroxyphenylpyruvate dioxygenase (HPPD), Photosystem II (PSII).

## Data Availability

All the data supporting the results presented in this article are included in the article.
